# Longitudinal analysis for the risk of depression according to the consumption of sugar-sweetened carbonated beverage in non-diabetic and diabetic population

**DOI:** 10.1038/s41598-023-40194-6

**Published:** 2023-08-09

**Authors:** Sung Keun Park, Yeongu Chung, Yoosoo Chang, Chang-Mo Oh, Jae-Hong Ryoo, Ju Young Jung

**Affiliations:** 1 Center for Cohort Studies, Total Healthcare Center, Kangbuk Samsung Hospital, Sungkyunkwan University School of Medicine, Seoul, Republic of Korea; 2 Department of Neurosurgery, Kangbuk Samsung Hospital, Sungkyunkwan University School of Medicine, Seoul, Korea; 3 Departments of Preventive Medicine, School of Medicine, Kyung Hee University, Seoul, Korea; 4 Departments of Occupational and Environmental Medicine, School of Medicine, Kyung Hee University, Seoul, Korea; 5 Total Healthcare Center, Kangbuk Samsung Hospital, Sungkyunkwan University, School of Medicine, 67, Sejong-daero, Jung-gu, Seoul, 04514 Republic of Korea

**Keywords:** Psychology, Medical research, Risk factors

## Abstract

Studies have presented that high intake of sugar-sweetened carbonated beverage (SSCB) was more associated with the prevalence of depression. However, longitudinal evidence is still insufficient to identify whether the effect of SSCB on incident depression is independent of metabolic factors. Therefore, to evaluate the effect of SSCB consumption on the risk of depression, we analyzed the risk of depression according to the consumption of SSCB in 87,115 working aged Koreans who responded to Center for Epidemiologic Studies Depression (CES-D) scale. They were categorized into 5 groups by SSCB consumption based on one serving dose (200 ml) with never/almost never, < 1 serving/week, 1 ≤ serving/week < 3, 3 ≤ serving/week < 5, and 5 ≤ serving/week. During follow-up, CES-D ≥ 16 was determined as incident depressive symptom. Cox proportional hazards model was used to calculate the multivariable-adjusted hazard ratio (HR) and 95% confidence intervals (CI) for depressive symptom. In analysis for all study participants, the risk of depressive symptom significantly increased proportionally to SSCB consumption (never/almost never: reference, < 1 serving/week: 1.12 [1.07–1.17], 1 ≤  ~  < 3 serving/week: 1.26 [1.19–1.33], 3 ≤  ~  < 5 serving/week: 1.32 [1.23–1.42], and ≥ 5 serving/week: 1.45 [1.33–1.59]). This association was identically observed in men, women, normal glycemic subgroup and prediabetes subgroup.

## Introduction

High intake of sugar-sweetened carbonated beverage (SSCB) is a major cause of global obesity epidemic^1^. The high intake of SSCB has been linked to a potential risk factor for cardiometabolic diseases^1–3^. The effect of SSCB may be mediated by large amount of sugar and high-fructose corn syrup that increase dietary glycemic load and serum triglyceride levels, leading insulin resistance (IR)^4,5^.

Depression is a common and serious medical illness with a lifetime prevalence ranging from approximately 11–15%^6^. Depression is a major public health problem as a leading global cause of increased disability-adjusted life years^7^. Therefore, it is clinically important to find out the modifiable risk factors for depression in terms of attenuating the burden of depression.

Epidemiological evidences have described the potential association between metabolic disorders, IR and depression. Obesity has the reciprocal relationship with depression, in which obesity can be a reason and consequence of depression^8,9^. Depressive disorders and their symptoms are associated with greater prevalence rate of metabolic syndrome^10^. There is evidence that the prevalence of depression is moderately increased in prediabetic patients and diabetic patients, compared to individuals with normal glucose metabolism^11^. IR has also been reported to associate with depression. A meta-analysis reported the weak but significant cross-sectional association between depressive symptoms and IR^12^. In a recent study, women with depressive symptoms had a 28.7% higher homeostasis model assessment-insulin resistance (HOMA-IR) level (*p* = 0.026), compared with women without depressive symptoms^13^. Thus, it can be speculated that high intake of SSCB may contribute to the development of depression via metabolic derangement and elevated IR. In fact, there have been studies displaying the potential association between SSCB consumption and the risk of depression^14,15^. However, it remains unclear whether higher consumption of SSBC is more associated with the risk of depression independent of glycemic status and IR. Moreover, longitudinal evidence is still insufficient to identify the role of SSBC consumption in the development of depression.

To obtain the insight for the effect of SSCB consumption on the development of depression, we longitudinally evaluated the risk depressive symptom according to the consumption of SSCB in working aged Korean adults. In addition, we conducted the subgroup analysis by glycemic status of study participants, which was to identify whether this association was impacted by metabolic factors.

## Result

Table 1 shows the clinical, biochemical, social characteristics of study subjects according to SSCB consumption. The mean age of the study subjects was 39.5 ± 6.8 years, and two-third of participants were male (64.2%, n = 55,941). Only one-third of subjects (28.9%, n = 25,246) consumed SSCB more than once a week. During 5.9 years of median follow-up period, 14.9% (n = 12,792) of study subjects fulfilled the definition of depressive symptom (CES-D ≥ 16). Group with most consuming SSCB (≥ 5 serving/week) was characterized by younger age and predominance of men. SSCB consumption ≥ 5 serving/week had the higher levels in fasting glucose, HOMA-IR, uric acid, BMI, alcohol consumption, total calorie intake, smoking, proportion of high education and prevalence of hypertension than other groups, despite modest numeric values in some cases. In contrast, SSCB consumption ≥ 5 serving/week had the lower levels in the proportion of high physical activity, the prevalence of diabetes mellitus (DM) and the proportion of marriage than never consumption group.

**Table 1:** Baseline clinical characteristics of study subjects according to the Sugar-sweetened carbonated beverage consumption.

Characteristics	Serving/week					
	Never	< 1	≥ 1 and < 3	≥ 3 and < 5	≥ 5	*P* for trend
Number	28,923	32,946	15,457	6004	3758	
Male sex (n, [%])	14,234 (49.2%)	20,908 (63.5%)	12,430 (80.4%)	5139 (85.6%)	3230 (85.3%)	< 0.001
Age (year)	41.7 ± 7.4	39.2 ± 6.4	37.8 ± 6.0	36.9 ± 5.7	36.9 ± 6.0	< 0.001
Fasting glucose (mg/dL)	96.2 ± 14.5	95.7 ± 13.2	96.2 ± 12.9	96.3 ± 14.3	97.0 ± 17.0	< 0.001
HbA1c (%)	5.7 ± 0.4	5.6 ± 0.4	5.6 ± 0.4	5.6 ± 0.5	5.6 ± 0.6	< 0.001
HOMA-IR	1.3 ± 1.0	1.4 ± 1.0	1.5 ± 1.0	1.5 ± 1.1	1.7 ± 1.9	< 0.001
Uric acid (mg/dL)	5.0 ± 1.4	5.4 ± 1.4	5.8 ± 1.4	6.0 ± 1.4	6.0 ± 1.4	< 0.001
BMI (kg/m^2^)	22.9 ± 3.1	23.3 ± 3.2	24.0 ± 3.2	24.2 ± 3.3	24.5 ± 3.4	< 0.001
Average alcohol use (g/day)	13.8 ± 22.5	14.6 ± 21.3	17.0 ± 22.1	18.2 ± 22.2	20.1 ± 28.1	< 0.001
Total calorie intake (kcal/day)	1485.2 ± 626.3	1576.5 ± 581.2	1765.4 ± 621.9	1934.8 ± 676.2	2228.7 ± 1116.9	< 0.001
Current smoker (n, [%])	5115 (17.7%)	7310 (22.2%)	4788 (31.0%)	2276 (37.9%)	1563 (41.3%)	< 0.001
High PA (n, [%])	6063 (21.0%)	5384 (16.3%)	2356 (15.2%)	939 (15.6%)	616 (16.1%)	< 0.001
Married (n, [%])	26,564 (91.8%)	29,301 (88.9%)	12,996 (84.1%)	4848 (80.7%)	2968 (78.4%)	< 0.001
High education (n, [%])	20,228 (69.9%)	24,479 (74.3%)	12,180 (78.8%)	4806 (80.0%)	2983 (77.6%)	< 0.001
Glycemic status (n, [%])						< 0.001
DM	1404 (4.9%)	1062 (3.2%)	477 (3.1%)	188 (3.1%)	133 (3.5%)	
Prediabetes	13,802 (47.7%)	15,670 (47.6%)	7184 (46.5%)	2755 (45.9%)	1774 (46.9%)	
Normal glycemia	13,717 (47.4%)	16,214 (49.2%)	7796 (50.4%)	3061 (51.0%)	1877 (49.6%)	
Hypertension (%)	3258 (11.3%)	3373 (10.2%)	1728 (11.2%)	667 (11.1%)	470 (12.4%)	< 0.001
CESD score	5.0 ± 4.1	5.0 ± 4.1	5.0 ± 4.2	5.1 ± 4.2	5.5 ± 4.2	< 0.001
Depressive symptom (n, [%])	3893 (13.5%)	4812 (14.6%)	2418 (15.6%)	979 (16.3%)	690 (18.2%)	< 0.001

Continuous variables are expressed as mean (± SD), and categorical variables are expressed as number (percentage (%)).

*BP* blood pressure, *BMI* body mass index, *PA* physical activity, *DM* diabetes mellitus, *CESD* Center for Epidemiologic Studies Depression.

The unadjusted and the multivariable adjusted HR and 95% CI for depressive symptom according to SSCB consumption are presented in Table 2. In fully adjusted analysis, compared with never/almost never consumption, the risk of depressive symptom increased proportionally to the consumption of SSCB (never/almost never consumption: reference, < 1 serving/week: 1.12 [1.07–1.17], 1 ≤  ~  < 3 serving/week: 1.26 [1.19–1.33], 3 ≤  ~  < 5 serving/week: 1.32 [1.23–1.42], and ≥ 5 serving/week: 1.45 [1.33–1.59]), *P* for trend < 0.001). This finding is reproduced in gender subgroup analysis. Although the incidence and incidence density was generally higher in women than men, the trends of association was similarly observed in both men and women (*P* for trend < 0.001).

**Table 2:** Hazard Ratio (HR) and 95% confidence intervals (CI) for depressive symptom (CESD ≥ 16) according to the Sugar-sweetened carbonated beverage consumption.

Characteristics	Serving/week					
	Never	< 1	≥ 1 and < 3	≥ 3 and < 5	≥ 5	*P* for trend
All participants (n)	28,923	32,946	15,457	6004	3785	
Unadjusted HR	1.00 (Reference)	1.06 (1.01–1.10)	1.13 (1.07–1.18)	1.18 (1.10–1.26)	1.35 (1.24–1.46)	< 0.001
Multivariable - adjusted HR	1.00 (Reference)	1.12 (1.07–1.17)	1.26 (1.19–1.33)	1.32 (1.23–1.42)	1.45 (1.33–1.59)	< 0.001
Incidence case [n, (%)]	3893 (13.5%)	4812 (14.6%)	2418 (15.6%)	979 (16.3%)	690 (18.2%)	
Incidence density/person year	27.0/144,338	28.5/169,102	30.3/79,875	31.7/30,884	36.2/19,085	
Men (n)	14,234	20,908	12,430	5139	3230	
Unadjusted HR	1.00 (Reference)	1.13 (1.06–1.20)	1.28 (1.20–1.37)	1.35 (1.24–1.47)	1.56 (1.42–1.72)	< 0.001
Multivariable- adjusted HR	1.00 (Reference)	1.13 (1.06–1.20)	1.26 (1.17–1.35)	1.30 (1.19–1.42)	1.45 (1.31–1.60)	< 0.001
Incidence case [n, (%)]	1577 (11.1%)	2695 (12.9%)	1816 (14.6%)	787 (15.3%)	559 (17.3%)	
Incidence density/person year	21.9/71,954	24.7/108,998	27.9/65,005	29.5/26,703	34.0/16,423	
Women (n)	14,689	12,038	3,027	865	555	
Unadjusted HR	1.00 (Reference)	1.10 (1.04–1.17)	1.27 (1.16–1.39)	1.46 (1.26–1.69)	1.55 (1.30–1.85)	< 0.001
Multivariable- adjusted HR	1.00 (Reference)	1.11 (1.05–1.18)	1.25 (1.14–1.37)	1.43 (1.24–1.67)	1.47 (1.23–1.76)	< 0.001
Incidence case [n, (%)]	2,316 (15.8%)	2,177 (18.1%)	602 (19.9%)	192 (22.2%)	131 (23.6%)	
Incidence density/person year	32.0/72,384	35.2/60,104	40.5/14,870	46.4/4,141	49.2/2,662	

Adjusting covariates: age, BMI, sex, physical activity, alcohol intake, hypertension, smoking, marital status, high education, total calorie intake, HOMA-IR.

Table 3 presents the subgroup analysis by glycemic status. In normal glycemic subgroups, the risk for depressive symptom significantly increased proportionally to the consumption of SSCB (never/almost never consumption: reference, < 1 serving/week: 1.09 [1.02–1.15], 1 ≤  ~  < 3 serving/week: 1.24 [1.15–1.34], 3 ≤  ~  < 5 serving/week: 1.26 [1.14–1.40], and ≥ 5 serving/week: 1.34 [1.19–1.52], *P* for trend < 0.001). This trend of association was identically observed in subjects with prediabetes (never/almost never consumption: reference, < 1 serving/week: 1.16 [1.08–1.23], 1 ≤  ~  < 3 serving/week: 1.28 [1.19–1.39], 3 ≤  ~  < 5 serving/week: 1.39 [1.25–1.55], and ≥ 5 serving/week: 1.58 [1.40–1.79], *P* for trend < 0.001). Despite statistical insignificance in some cases, diabetes subgroups showed the similar association (never/almost never consumption: reference, < 1 serving/week: 1.20 [0.95–1.49], 1 ≤  ~  < 3 serving/week: 1.32 [0.99–1.73], 3 ≤  ~  < 5 serving/week: 1.35 [0.92–1.99], and ≥ 5 serving/week: 1.56 [1.004–2.43], P for trend = 0.032).

**Table 3:** Hazard Ratio (HR) and 95% confidence intervals (CI) for depressive symptom (CESD ≥ 16) according to the Sugar-sweetened carbonated beverage consumption in subgroups stratified by glycemic status.

Characteristics	Serving/week					
	Never	< 1	≥ 1 and < 3	≥ 3 and < 5	≥ 5	*P* for trend
Normal glycemia (n)	13,717	16,214	7796	3061	1877	
Unadjusted HR	1.00 (Reference)	1.01 (0.96–1.08)	1.08 (1.01–1.16)	1.10 (1.00–1.22)	1.20 (1.07–1.35)	< 0.001
Multivariable- adjusted HR	1.00 (Reference)	1.09 (1.02–1.15)	1.24 (1.15–1.34)	1.26 (1.14–1.40)	1.34 (1.19–1.52)	< 0.001
Incidence case [n, (%)]	1961 (14.3%)	2404 (14.8%)	1234 (15.8%)	490 (16.0%)	327 (17.4%)	
Incidence density/person year	28.5/68,874	28.8/83,468	30.7/40,209	31.3/15,656	34.2/9572	
Prediabetes (n)	13,802	15,670	7184	2755	1775	
Unadjusted HR	1.00 (Reference)	1.10 (1.03–1.17)	1.16 (1.08–1.25)	1.25 (1.13–1.39)	1.49 (1.33–1.68)	< 0.001
Multivariable- adjusted HR	1.00 (Reference)	1.16 (1.08–1.23)	1.28 (1.19–1.39)	1.39 (1.25–1.55)	1.58 (1.40–1.79)	< 0.001
Incidence case [n, (%)]	1762 (12.8%)	2253 (14.4%)	1107 (15.4%)	456 (16.6%)	337 (19.0%)	
Incidence density/person year	25.6/68,895	28.0/80,538	29.6/37,365	32.0/14,252	37.9/8884	
Diabetes mellitus (n)	1404	1062	477	188	133	
Unadjusted HR	1.00 (Reference)	1.18 (0.95–1.47)	1.29 (0.99–1.69)	1.37 (0.94–1.99)	1.60 (1.06–2.41)	0.010
Multivariable- adjusted HR	1.00 (Reference)	1.20 (0.95–1.49)	1.32 (0.99–1.73)	1.35 (0.92–1.99)	1.56 (1.004–2.43)	0.032
Incidence case [n, (%)]	170 (12.1%)	155 (14.6%)	77 (16.1%)	33 (17.6%)	26 (19.5%)	
Incidence density/person year	25.9/6569	30.4/5097	33.4/2302	35.3/936	41.4/629	

Adjusting covariates: age, BMI, sex, physical activity, alcohol intake, hypertension, smoking, marital status, high education, total calorie intake, HOMA-IR.

## Discussion

Through a longitudinal analysis for 87,115 Koreans with working age of 39.5 ± 6.8 years, we demonstrated that the elevation of SSCB consumption was significantly associated with the increased risk of depressive symptom with dose–response pattern. This association was independent of metabolic derangements such as obesity, IR and DM that account for the major pathophysiology of SSCB. In particular, dose–response relationship was identically observed in all of subgroup analyses for gender and glycemic status. These results support the hypothesis that high intake of SSCB provokes the development of depression.

There have been epidemiological reports being line with ours. Cross-sectional studies have suggested the potential association between SSCB consumption and depression. In the multivariate analysis for 4741 Australian, those who consumed more than half a liter of soft drink per day had approximately 60% greater risk of having depression, stress-related problem, suicidal ideation, or mental problems, compared with those not consuming soft drinks^16^. A study for 3667 Chinese adults showed that the odds ratios for elevated depressive symptoms increased proportionally to the levels of soft drink consumption, even after adjusting for potential confounders^17^. A recent meta-analysis for 5 cross-sectional studies showed that the relative risk of depression for the highest versus lowest consumption of SSBs was 1.38 (95% CI 1.26–1.52)^15^. However, results obtained from cross-sectional studies are limited to prove causative relationship between factors. Moreover, there is a possibility that people with depressive symptom tend to crave sweet beverages. Therefore, longitudinal analysis may be helpful to investigate causative relationship, attenuating the bi-directional effect. Nonetheless, results from longitudinal analysis were inconsistent as well as less substantial to identify the effect of SSCB consumption on the development of depression. In a prospective study for 263,923 US adults, Guo et al., indicated that subjects with consuming ≥ 4 cans/cups of soft drinks per day had the higher risk of depression than non- consumers with odd ratio of 1.30 (95% CI 1.17–1.44)^18^. However, their study didn’t show the dose-dependent relationship between consumption of soft drink and the risk of depression. Prospective findings from the Whitehall Study II presented that the odd ratio for recurrent depression increased in the highest tertile groups of sweet beverage consumption in the 23,245 British, but lost statistical significance after adjusting the diet-related factors (1.47 [0.98–2.22])^19^. In a longitudinal analysis for 15,546 from Spanish university graduates, although the highest quartile of added sugars consumption significantly increased the risk of depression, the association between sugar-sweetened beverage consumption and the risk of depression was not found^20^. Our study is differentiated from previous longitudinal analyses in that our analysis showed the clear dose–response pattern of relationship between SSCB consumption and the risk of depression. Our study may provide additional evidence to prove the harmful effect of SSCB consumption on the mental health.

Plausible mechanisms link SSCB consumption to depression may include metabolic derangements like obesity, IR and DM. However, our results indicate that the dose–response relationship between SSCB consumption and the risk of depression is independent of BMI, HOMA-IR, and total calorie intake. Additionally, this association was maintained even in glycemic subgroup analyses. Thus, our findings suggest that the effect of SSCB on depression may be partly independent of metabolic derangements. A potential explanation for these findings may be the detrimental effect of elements rich in SSCB on neurobiological system. SSCB contains large amount of sugar and fructose corn syrup. Laboratory evidence from rat model showed that high consumption of fructose during preadolescence showed increased anxiety-like behavior and depressive-like behavior in their adulthood^21^. High fructose corn syrup -Moderate Fat diet induced changes in the gut microbiota and neuroactive metabolites, which contribute to maladaptive alterations in ventral striatal function underlying neurobehavioral impairment^22^. Overconsumption of added sugar has been hypothesized to increase the reactivity of hypothalamic–pituitary–adrenal axis leading to the dysregulation of stress response^23^. In addition, high consumption of SSCB result in obesity and DM, which promotes chronic inflammation and insulin resistance (IR). Previous studies have demonstrated that chronic inflammation and IR have a potential role in the development of depression^24,25^. Nonetheless, it still remains unclear in the mechanism that mediates SSCB consumption with depression, and warrants the further studies to examine the underlying mechanisms.

Depression is more prevalent in women than men. A meta-analysis indicated that the global 12-month prevalence of major depressive disorder was 5.8% in females and 3.5% in males^26^. In contrast, previous cohort studies have shown that men tended to more consume soft drink than women. Our study participants were also characterized by predominant prevalence of depression in women and higher consumption of SSCB in men^18–20^. Previous studies have displayed gender difference in the association between sweet beverage and depression^18–20^. Differences in the sample sizes among studies and in the pathways of depression by gender were raised as plausible explanations for gender difference^19^. Thus, we were to verify gender difference in the effect of SSCB consumption on the risk of depression. Interestingly, the dose–response relationship between SSC consumption and the risk of depressive symptom was reproduced in both men and women. This finding implies that the harmful effect of SSCB on depression is independent of overall consumption of SSCB and prevalence of depression. In particular, considering that our study participants were relatively young with age of 39.5 ± 6.8, it is postulated that the adverse effect of SSCB consumption on mental health can begin at an early age. Thus, our results may be evidence to recommend abstaining from SSCB in young age.

### Our study has several limitations

First, the amount of SSCB intake is relatively smaller than that in other countries. In a study for U.S adults, the amount of soft drink associated with depression was ≥ 4 cans/cups per a day. This amount seems to be higher than the highest consumption of SSCB in our study (≥ 5 servings of 200 ml SSCB per a week). Therefore, our findings are not likely to be extrapolated into other nations. Second, most of our study subjects were apparently healthy and working aged adults. Our results are not likely to be generalized to general population including the elderly and adolescent. Third, our data didn’t include the information about low-energy carbonated beverages (diet soda) or noncarbonated sugar-sweetened beverages. There was a report that diet soft drink was more associated with depression than regular soft drink^18^. Further studies should prospectively investigate the risk of depression according to the more specified category of beverage in larger and more generalized sample.

In conclusion, our study indicated the dose–response relationship between SSCB consumption and the risk of depressive symptom independent of metabolic factors in working aged Korean adults. This association was identically observed in subgroup analyses for gender and glycemic status. Our results may be additional evidence for the harmfulness of SSCB, supporting the policy or social campaign to limit marketing of SSCB.

## Methods

### Study population

Relevant clinical and echocardiographic data were obtained from Kangbuk Samsung Health Study (KSHS). KSHS is a cohort study to investigate the medical data of Koreans who have received medical health check-up in Kangbuk Samsung Hospital. Korea’s Industrial Safety and Health law orders that all of Korean employees should receive medical health check-up annually or biennially.

**Figure 1:**
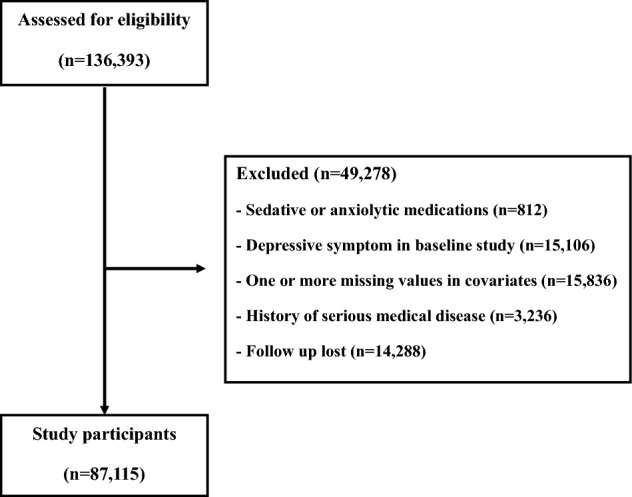
Flow chart of enrolled study participants.

Among study participants in KSHS, we initially enrolled 136,393 subjects who had responded to semi-quantitative food frequency questionnaire (FFQ) and Center for Epidemiologic Studies Depression (CES-D) between March 2011 and December 2012. Among these subjects, we excluded 812 subjects with taking sedative or anxiolytic medications and 15,106 subjects with depressive symptom in baseline analysis. Furthermore, 15,836 subjects with missing value in covariate data (e.g. BMI, hypertension, alcohol intake) and 3236 subjects with a history of serious medical diseases (e.g. coronary heart disease, stroke, and cancer) were further excluded. Additionally, 14,288 subjects with lost to follow-up were excluded. Finally, the total number of eligible study participants was 87,115 (Fig. 1). The median period of follow-up was 5.9 years.

Ethics approvals for the study protocol and analysis of the data were obtained from the institutional review board (IRB) of Kangbuk Samsung Hospital (IRB No. KBSMC 2020-09-25). All procedures performed in studies involving human participants were in accordance with the ethical standards of the IRB of the Kangbuk Samsung Hospital and with the 1964 Helsinki declaration and its later amendments or comparable ethical standards. IRB of Kangbuk Samsung Hospital approved the exemption of informed consent for the study because we only assessed retrospective data with de-identified personal information obtained from routine health check-up.

### Clinical and biochemical data collection

Study data included medical history assessed by self-administered questionnaire, anthropometric measurements and laboratory measurements. All study subjects were asked to respond to a health-related behavior questionnaire, which included the topics of alcohol consumption, smoking and exercise. The degree of physical activity was evaluated by the Korean-validated version of the International Physical Activity Questionnaire (IPAQ) short form (SF)^27^. High physical activity was defined on the basis of health-enhancing physically active in IPAQ as follows:^1^ vigorous intensity activity on three or more days per week accumulating 1500 MET-min/week,^2^ 7 days of any combination of walking, moderate intensity, or vigorous intensity activities achieving at least 3000 MET-min/week. Subject with high education were defined as those who had a university degree or higher. Hypertension was defined as a prior diagnosis of hypertension or having a measured BP ≥ 140/90 mmHg at initial and follow up examinations. Trained nurses measured BP on sitting position by automatic device (53,000-E2, Welch Allyn, USA) three times after a 5 min rest with at least 30 s interval. Final BP levels were obtained as average of second and third BP measurements. The BMI was calculated by dividing weight (kilograms) by square of height (meters^2^).

Blood samples were collected after more than 12 h of fasting and were drawn from an antecubital vein. The fasting serum glucose was measured using the hexokinase method, and hemoglobin A1c (Hba1c) was measured using an immunoturbidimetric assay with a Cobra Integra 800 automatic analyzer (Roche Diagnostics, Basel, Switzerland). Serum uric acid levels were measured enzymatically using an automatic analyzer Advia 1650 Autoanalyzer, Bayer Diagnostics; Leverkusen, Germany).

Glycemic status was classified into normal glycemia, prediabetes and DM. DM was defined as one of following conditions; fasting glucose ≥ 126 mg/dL, hemoglobin A1 c (HbA1c) ≥ 6.5%, and a prior diagnosis of DM^28^. Fasting glucose of 100–125 mg/dl or HbA1c of 5.7–6.5% were regarded as prediabetes. Insulin resistance was evaluated by calculating homeostasis model assessment-insulin resistance (HOMA-IR) as following formula: HOMA-IR = fasting serum insulin (uU/ mL) × fasting serum glucose (mg/dl)/405^29^.

### Assessment of FFQ data

We assessed the dietary intake of KSHS participants using the FFQ that was developed for the Korean genome epidemiologic study. The dietary data to design the FFQ were obtained from the Korea Health and Nutrition Examination Survey^30,31^. A detailed description of the FFQ^30^ and its validation in the Korean population has been described in a previous study^31^. The food consumption frequency was composed of nine categories (e.g., SSCB intake was categorized never or rarely, once a month, two or three times a month, once or twice a week, three or four times a week, five or six times a week, one or two times a day, three or four times a day, and more than five times a day) and three serving sizes for each food (e.g., SSCB consumption was categorized as 0.5, 1 and 2 serving. 1 serving = 200 ml). Food photographs with usual intake portions also were included to increase the understanding and study reliability in study subjects. All subjects categorized into five group according to SSCB consumption as follows: never/almost never, < 1 serving/week, 1 ≤  ~  < 3 serving/week, 3 ≤  ~  < 5 serving/week, and ≥ 5 serving/week) Total energy and nutrient intake was calculated by the Can-Pro 3.0 software developed by The Korean Nutrition Society^32^.

### Assessment of depressive symptom

Depressive symptoms were assessed using the Korean versions of CES-D scale^33^. The CES-D is a self-report questionnaire designed to assess the current presence of depressive symptoms in the general population^34^. We used the 4-factors 20-items CES-D Scale with scores ranging from 0 to 3, with 0 indicating that the depressive symptom was experienced rarely and 3 indicating that the symptom was experienced most of the time in the past week. (e.g. “I thought my life had been a failure.” 0 = seldom (not at all or less than 1 day), 1 = sometimes (1–2 days), 2 = often (3–4 days), 3 = almost always (5–7 days)). This scale has been widely used across the world and shown the validity and reliability in the Korean general population^33^. Depressive symptom was defined in the total score of CES-D ≥ 16. Therefore, in baseline analysis, subjects with CES-D ≥ 16 were regarded as the presence of depressive symptom in baseline and excluded from study participants. During follow-up, the subjects who newly fulfilled CES-D ≥ 16 were determined as the cases of incident depressive symptom. Detailed descriptions of study population and data collection have been included in previous studies^35^.

### Statistical analysis

The baseline parameters with five SSCB intake groups are presented as means ± standard deviation for continuous variables and as proportions for categorical variables. The linear regression model was used for continuous variables and Cochran-Armitage trend test was used for categorical variable to assess linear response between SSCB consumption and biochemical, health related behavior, and chronic disease.

A Cox proportional hazards model was used to calculate the age-adjusted and multivariable-adjusted hazard ratio (HR) for depressive symptom and their 95% confidence intervals (CI) in each study groups (multivariable adjusted HR [95% CI]). The models were adjusted for multiple covariates including age, BMI, sex, physical activity, alcohol intake, hypertension, smoking, marital status, total calorie intake, and HOMA-IR. The covariates of the multivariable model were selected for the presence of significant differences between groups or established risk factors for depression. The incidence cases, incidence density (incidence cases per 1000 person-years), person years of each group were calculated. Trend analysis was conducted using the median of range in each SSCB consumption category (0 in never/almost never, 0.5 in < 1 serving/week, 2 in 1 ≤  ~  < 3 serving/week, 4 in 3 ≤  ~  < 5 serving/week, and 7 in > 5 serving/week). Subgroup analyses were conducted in gender and glycemic status subgroup.

The All statistical analyses were performed using R 3.6.3 (R Foundation for Statistical Computing, Vienna, Austria), and a value of *P* < 0.05 (two-sided) was considered statistically significant in all analyses.

## Data Availability

The data that support the findings of this study are available from Kangbuk Samsung Cohort Study, but restrictions apply to the availability of these data, which were used under license for the current study, and so are not publicly available. Data are however available from the authors upon reasonable request and with permission of Kangbuk Samsung Cohort Study.
